# Effects of Two Decellularization Protocols on the Mechanical Behavior and Structural Properties of the Human Urethra

**DOI:** 10.3390/ijms252212361

**Published:** 2024-11-18

**Authors:** Marcela Kuniakova, Zuzana Varchulova Novakova, Daniel Haspinger, Justyna Anna Niestrawska, Martin Klein, Paulina Galfiova, Jan Kovac, Michal Palkovic, Lubos Danisovic, Niels Hammer, Stanislav Ziaran

**Affiliations:** 1 Institute of Medical Biology, Genetics and Clinical Genetics, Faculty of Medicine, Comenius University in Bratislava, Sasinkova 4, 811 08 Bratislava, Slovakia; marcela.kuniakova@fmed.uniba.sk (M.K.); zuzana.varchulova@fmed.uniba.sk (Z.V.N.); jan.kovac@nurch.sk (J.K.); 2 Division of Macroscopic and Clinical Anatomy, Gottfried Schatz Research Center, Medical University of Graz, Auenbruggerplatz 25, A-8036 Graz, Austria; daniel.haspinger@medunigraz.at (D.H.); justyna.niestrawska@medunigraz.at (J.A.N.); niels.hammer@medunigraz.at (N.H.); 3 Institute of Histology and Embryology, Faculty of Medicine, Comenius University in Bratislava, Sasinkova 4, 811 08 Bratislava, Slovakia; martin.klein@fmed.uniba.sk (M.K.); paulina.galfiova@fmed.uniba.sk (P.G.); 4 National Institute of Rheumatic Diseases, Nabr. I. Krasku 4, 921 12 Piestany, Slovakia; stanislav.ziaran@fmed.uniba.sk; 5 Institute of Pathological Anatomy, Faculty of Medicine, Comenius University in Bratislava, Sasinkova 4, 811 08 Bratislava, Slovakia; michal.palkovic@fmed.uniba.sk; 6 Department of Orthopedic and Trauma Surgery, University of Leipzig, Liebigstraße 20, 04103 Leipzig, Germany; 7 Division of Biomechatronics, Fraunhofer Institute for Forming Tools, 01187 Dresden, Germany; 8 Department of Urology, Faculty of Medicine, Comenius University in Bratislava, Limbova 5, 833 05 Bratislava, Slovakia

**Keywords:** urethra, decellularization, morphology, biomechanical properties, tissue engineering

## Abstract

This study evaluates the effects of two decellularization protocols, enzyme-detergent (ED) and detergent-detergent (DD), on the structural and biomechanical properties of human urethral tissue. Urethral samples from 18 individuals were divided into ED (*n* = 7) and DD (*n* = 11) groups, with native samples (*n* = 3) serving as controls. Histological and ultrastructural analyses confirmed that both protocols effectively removed cellular content while preserving essential extracellular matrix (ECM) elements, such as collagen and elastic fibers. Immunohistochemical staining for collagen IV and fibronectin revealed no significant differences between decellularized and native tissues, indicating intact ECM structure. Biomechanical testing demonstrated that DD-treated tissues had significantly lower Cauchy stress (1494.8 ± 518.4 kPa) when compared to native tissues (2439.7 ± 578.7 kPa, *p* = 0.013), while ED-treated tissues were similar to both groups. Both decellularized groups exhibited reduced stretch at failure and elastic modulus compared to native tissues. Cytotoxicity assays using adipose-derived stem cells demonstrated no signs of toxicity in either protocol. Overall, both ED and DD protocols effectively preserved the urethral ECM structure and mechanical properties, making them suitable for potential use in tissue-engineered grafts and for biobanking purposes. Further research is needed to refine and optimize decellularization methods to improve scaffold recellularization and ensure clinical safety and efficacy.

## 1. Introduction

The urethra can be affected by various factors that lead to the narrowing of the urethral lumen, resulting in obstruction of urine flow. This can significantly impact quality of life and, in severe cases, may lead to permanent organ impairment. Urethral scarring causes narrowing of the lumen, manifesting as a urethral stricture. Scar tissue has inferior biological and biomechanical properties and contributes to the reduction in the urethral lumen, leading to the development of lower urinary tract symptoms (LUTS) [1]. Patients with a urethral stricture often experience recurrent urinary tract infections and progression to acute urinary retention [2]. The incidence of urethral strictures in males over 55 is approximately 1%, with actual rates varying by geographic location, income, and socioeconomic factors [3].

Various surgical techniques, including urethral dilation and direct vision internal urethrotomy (DVIU), have been developed to treat urethral strictures. DVIU success rates vary widely, from 8 to 80%, depending on patient selection, follow-up length, and other factors [4,5,6,7]. However, the long-term success rates of these techniques are low, with a relatively high risk of recurrence and patency rates between 8% and 77% for DVIU [4]. Longer strictures are more likely to recur, with a recurrence risk within 12 months as high as 40% for strictures shorter than 2 cm, 50% for those between 2 and 4 cm, and 80% for strictures longer than 4 cm. Thus, stricture length is the main predictor of recurrence. Complications from internal urethrotomy are more likely in men with a history of urethral trauma, multiple stricture segments, and long (>2 cm) strictures [8]. Consequently, longer strictures often require open multistage urethroplasty with lingual or buccal grafts or skin flaps to replace deficient functional tissue [9]. Despite a high recurrence-free and patency rate of 90.5% [10], buccal or lingual graft harvesting carries risks [11,12]. Additionally, non-hairy skin from patients with lichen sclerosis may have limitations [13,14], as fibroblasts in lichen sclerosis-affected skin produce excess collagen, potentially worsening urethral narrowing and strictures [15].

Tissue engineering (TE) offers a promising alternative to traditional urethroplasty for addressing urethral defects. This approach focuses on creating functional biocompatible scaffolds that mimic the architecture and composition of the native extracellular matrix (ECM), essential for successful tissue regeneration. In TE-based urethral reconstruction, these scaffolds serve as structural and functional platforms for cellular attachment, proliferation, and differentiation. The integration of various cell types, including stem and urothelial cells, enhances regenerative potential, promoting the development of functional tissue that closely resembles the native urethra [16]. In recent years, numerous biomaterials have been explored as scaffold candidates, including natural biomaterials such as collagen, chitosan, gelatin, elastin, and cellulose, known for their excellent biocompatibility and bioactivity [17,18,19]. Synthetic polymers like polylactic acid (PLA), polyglycolic acid (PGA), poly(lactic-co-glycolic acid) (PLGA), and polyhydroxybutyrate (PHB) have also gained attention for their controllable degradation rates and mechanical properties. Acellular tissue matrices, derived from decellularized native tissues, offer an attractive option by retaining the ECM’s structural integrity and composition [20,21,22]. However, despite these advancements, challenges remain in creating engineered tissue constructs capable of supporting the long-term repair of extensive urethral strictures. Achieving the optimal balance between scaffold mechanical properties, degradation rates, and healthy tissue regeneration is essential for developing durable functional urethral replacements. Further research is needed to refine these scaffolds and fully realize TE’s potential in clinical applications for urethral reconstruction.

In this article, we provide a detailed investigation into the effects of two specific decellularization protocols on the mechanical and structural properties of acellular tissue matrices, which can serve as scaffolds for cells in TE and regenerative medicine. Furthermore, the development of acellular tissue matrices has significant implications for biobanking, enabling long-term storage of biologically relevant materials that can be re-seeded with patient-specific cells for future regenerative applications.

## 2. Results

### 2.1. Evaluation of the Urethra General Structure and ECM Components

**Figure 1:**
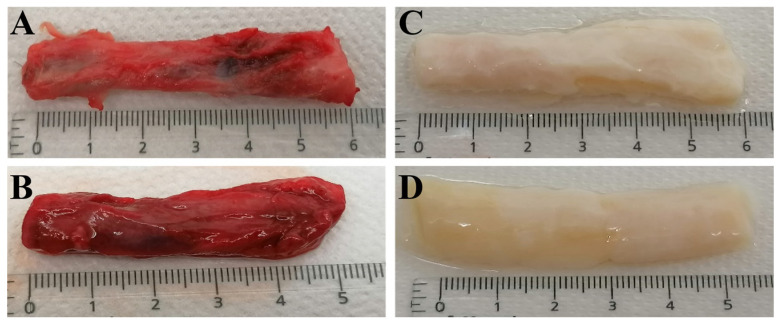
Macroscale appearance of the human urethral tissue: (**A**,**B**) urethral tissue prior to the decellularization process; (**C**) urethral tissue post-ED protocol decellularization; (**D**) urethral tissue post-DD protocol decellularization.

The initial evaluation of decellularization efficacy was based on macroscopic appearance. Native urethral tissue is typically rosy red (Figure 1A,B). Decellularization using protocol ED or protocol DD resulted in a pale almost translucent white color (Figure 1C,D), indicating cell removal. In rare cases, a less pronounced color change was observed in the mid portion of the sample. However, histological analysis demonstrated no different level of decellularization in these areas.

Light microscopic evaluation of the native specimens stained with hematoxylin and eosin (HE) revealed a typical structure of the male spongy urethra consisting of stratified columnar epithelium with urethral lacunas of Morgagni. Under the epithelium, connective tissue lamina propria was present with immune cells, fibroblasts, collagen and elastic fibers, blood, and lymphatic vessels. Under the mucosa was a double smooth muscle layer consisting of inner longitudinal and outer circular layers. We also observed the outermost connective tissue adventitia. Around the spongy urethra, the corpus spongiosum penis was found with trabeculae separating it into many irregular sponge-like cavities. When harvesting the necropsy specimens of the spongy urethra, it is tough to separate the urethra from its surroundings ideally. Therefore, both control and experimental specimens contained the corpus spongiosum to various extents, even though it is not a part of the spongy urethra wall.

**Figure 2:**
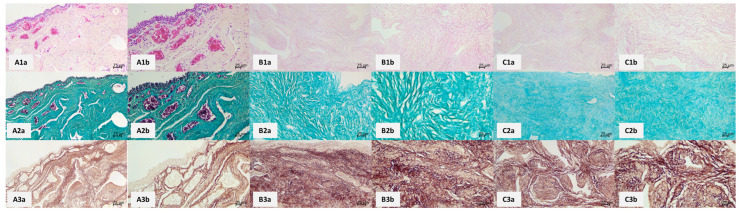
Histological structure of a representative panel of human spongy urethra as seen in control and decellularized samples using ED and DD protocols. HE staining of the control samples. (**A1a**,**A1b**) demonstrate the normal structure of the human spongy urethra with stratified columnar epithelium, connective tissue (lamina propria) with blood vessels, lymphatic vessels, collagen, and elastic fibers (normal structure of collagen and elastic fibers were best seen using Masson green trichrome staining and orcein, respectively). Under the lamina propria, there are two layers of smooth muscle. The outermost layer of the urethra is the connective tissue adventitia. Outside, the urethra blends with the tissue of the corpus spongiosum. (**A2a**,**A2b**) depict the control sample stained with Masson green trichrome, which shows the elaborate network of collagen fibers. (**A3a**,**A3b**) capture orcein-stained control samples for elastic fibers visualization. (**B1a**–**B3b**) are photomicrographs of decellularized experimental samples according to the ED protocol. The pink color in HE-stained samples (**B1a**,**B1b**) has diminished saturation. The main reason is the loss of cellular components with eosinophilic (pink) cytoplasm. Collagen fibers (**B2a**,**B2b**) have no visible changes in structure or number compared to controls. The same applies to the orcein-stained samples (**B3a**,**B3b**). (**C1a**–**C3b**) show the photomicrographs of samples decellularized according to the DD protocol. Their characteristics are virtually the same as in the ED protocol, namely a slightly lower saturation in HE-stained samples (**C1a**,**C1b**) and normal density and structure of collagen (**C2a**,**C2b**) and elastic (**C3a**,**C3b**) fibers.

**Figure 3:**
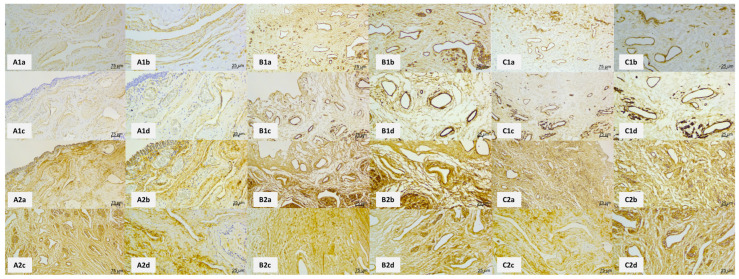
Immunohistochemical visualization of a representative panel of human spongy urethras as seen in control (**A**) and decellularized samples using ED (**B**) and DD (**C**) protocols. Immunohistochemical visualization of collagen IV in control samples (**A1a**–**A1d**) shows that collagen IV is found mainly around blood vessels and under the epithelial lining since it is an integral component of basal laminae. ED protocol of decellularization did not have any detrimental effect on collagen IV, evidenced by unaltered collagen IV positivity (**B1a**–**B1d**). The same is true for the DD protocol (**C1a**–**C1d**)—there is no observable change in collagen IV distribution after the decellularization process. (**A2a**–**A2d**) show fibronectin positivity in the control samples. Fibronectin is widely distributed in the samples since it is a multiadhesive glycoprotein vital for ECM integrity. (**B2a**–**B2d**) capture the fibronectin positivity in ED protocol samples. There is a lack of any detectable change in fibronectin distribution. The same applies to DD protocol samples (**C2a**–**C2d**)—the fibronectin positivity is ubiquitous, as seen in controls.

Figure 2 compares control and decellularized specimens according to the ED and DD protocols. Compared to controls, ED and DD protocols eliminated all cellular components. A lack of purple nuclei indicated the total absence of epithelial lining. In the connective and muscle tissue, all cell nuclei were gone, and only the components of the ECM persisted. Using Masson green trichrome staining, there was no evident difference in the structural characteristics of collagen fibers between the controls and experimental samples of either group. The orcein staining used to visualize elastic fibers was also evaluated, revealing that the density of elastic fibers was comparable between controls and both experimental specimens. Immunohistochemical staining (IHC) against collagen IV and fibronectin was used to compare the positivity of these two crucial ECM components (Figure 3). Collagen IV, which is a component of basal laminae, and fibronectin, which is vital for ECM structural and functional integrity, showed no significant differences between the controls and both experimental groups. Collagen IV was observed mainly surrounding the blood vessels, whereas fibronectin was ubiquitous throughout the entire ECM compartment. In summary, the light microscopic examination was adequate for evaluating proper cellular removal by decellularization. It also has not revealed any detrimental morphological changes to ECM by the decellularization process.

### 2.2. Ultrastructural Changes Assessed by SEM

**Figure 4:**
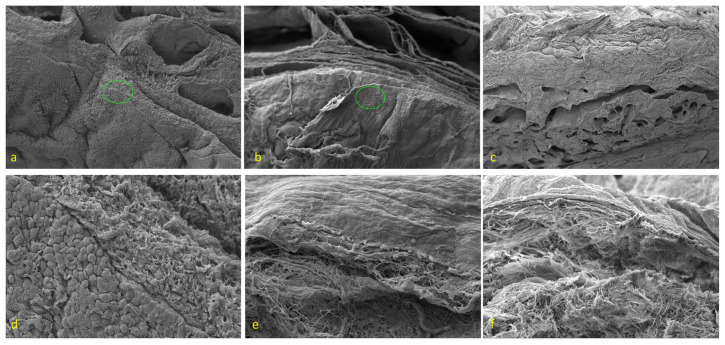
Scanning electron microscopy representative panel. (**a**) A topography of native sample in overview. Mag ×104. Left bottom displays a sheet of stratified columnar epithelium, attached to the basal lamina, a layer continuation with underlying connective tissue. Vascular spaces of the adjacent corpus spongiosum in the field of the cutline surface. The green circle is the region of interest (ROI) described in (**d**). (**b**) A topography of ED-processed sample in the overview. Mag ×89. None of the epithelium faces the lumen of the urethra. No detectable changes in ECM architecture at the overview level. The green circle is the (ROI described in (**c**,**e**) A topography of DD-processed sample in the overview. Mag ×80. None of the epithelium faces the lumen of the urethra. The minimal changes in ECM architecture at the overview level. (**d**) A detail of the encircled ROI of the native sample shows a borderline of the epithelium and the lamina propria with ECM. A top view of superficial columnar cells is displayed in a polygonal shape. Epithelial cells firmly adhere to the basal lamina. Mag ×596. (**e**) A detail of the encircled ROI shows a topography of decellularized mucosa using the ED protocol. No epithelium is evident; the naked lamina basalis reflects the smooth surface and the collagen fibers in the ECM almost show a natural architecture. Mag ×787. (**f**) A detail of the topography of decellularized mucosa using the DD protocol. No epithelium, the smooth lamina basalis, and a mild untangling of collagen bundles just beneath. Mag ×700.

The SEM evaluation visualized the changes in both spatial topography and the ultrastructure, comparing control and experimental decellularized samples, regarding the structural content beyond the resolving power of the light microscope. Micrographs displayed a topography of the human urethra´s luminal surface and the adjacent supporting connective tissue components (Figure 4). The samples of two different protocols have been compared using a three-dimensional SEM observation. The native urethra (a control sample) is depicted at the superficial layer of distinct stratified columnar epithelium (Figure 4a,b), which faces the lumen of the urethra. The columnar cells were tightly packed in the form of a sheet and exhibited polygonal-shaped topography. A lamina basalis, the structural site for the basal epithelial cell attachment, was not evident, masked by epithelial lining (Figure 4b). The surface view of the decellularized samples confirmed epithelial desquamation due to the process used by both mentioned protocols. No cells of the epithelium remained on the luminal surface of the urethra (Figure 4d,e). The decellularization process resulted in epithelial desquamation and revealed a topography of the naked lamina basalis. The lamina basalis resisted a sample procedure and its ultrastructure reflected the smooth compact uniform layer (Figure 4e). The content of the lamina propria, a layer beneath the epithelium, was uncovered due to tissue harvesting (side view of cutline surface). The same was applicable to the adjacent connective tissue, including vascular cavernae of the corpus spongiosum (Figure 4a,d). The ECM of the lamina propria in control samples displayed collagen fibers in a wave-like bundle arrangement, running in more directions. No evident cells of loose connective tissue were identified between the collagen fibers, due to the cell shape in the meaning of topography (Figure 4c). The experimental decellularized sample components of the ECM persisted with minimal structural changes. No relevant disarrangement of collagen fibers or tissue damage was noticed in detail observation (Figure 4e). SEM confirmed the structural resistance of tissue components in decellularization procedures, regardless of the two different protocol approaches; the initial evaluation of decellularization efficacy was based on the macroscopic appearance.

### 2.3. DNA Detection

**Figure 5:**
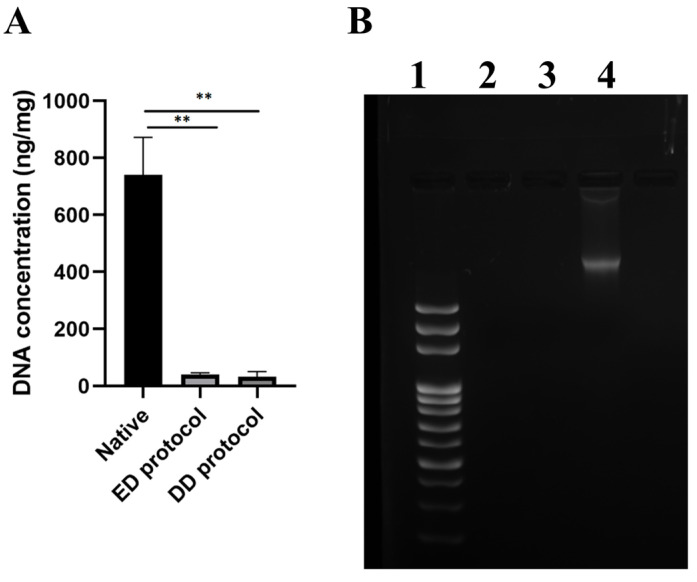
Qualitative and quantitative DNA content of native and decellularized human urethra evaluating the effectiveness of the decellularization process to remove cell components. (**A**) Quantification of DNA in native and decellularized samples (ng/DNA per mg/tissue dry weight). Results as mean ± SD, n = 18, ** *p* ≤ 0.001, *t*-test; (**B**) Qualitative fragment length analysis of genomic DNA in 1.5% agarose gel. 1—length ladder, 2—ED protocol decellularized sample, 3—DD protocol decellularized sample, 4—native urethra sample.

Besides the absence of cell nuclei, two other criteria are essential for successful decellularization: the amount of DNA should not exceed 50 ng per mg of dry tissue weight and the remaining DNA should not exceed 200 bp. The DNA content in native urethras was 715.4 ± 134.1 ng/mg tissue; the residual DNA in ED decellularized samples was 38.1 ± 7.6 ng/mg tissue and 31.2 ± 14.3 ng/mg tissue in DD decellularized samples (Figure 5A). A significant difference in residual DNA levels (*p* < 0.001; *n* = 18) was confirmed by statistical analysis. Gel electrophoretic analysis revealed intact DNA bands above 3000 bp in native urethra samples; in the decellularized samples, the analysis confirmed a significant removal of DNA (Figure 5B).

### 2.4. Biomechanical Properties

**Figure 6:**
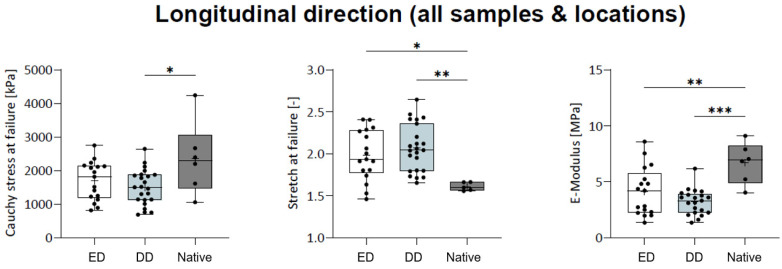
Longitudinal urethra load–deformation properties. Longitudinal urethra load–deformation properties are location and acellularization-protocol dependently. (*—*p* ˂ 0.05; **—*p* ˂ 0.01; ***—*p* ˂ 0.001).

For the combined longitudinal load–deformation trials, Cauchy stress was significantly lower in the DD group (1494.8 ± 518.4 kPa) when compared to the native group (2439.7 ± 578.7 kPa; *p* = 0.013). The ED group (1693.0 ± 568.7 kPa) was no different from the DD and native groups, respectively (Figure 6). Stretch at failure was significantly lower between the native state (1.98 ± 0.29) and ED group (2.07 ± 0.28; *p* = 0.030) or the (DD 1.67 ± 0.14) when compared to the ED group (*p* = 0.005), respectively, being no different between the ED and DD groups. Emod was significantly lower in the ED group (4.13 ± 2.09 MPa; *p* = 0.007) and in the DD group (3.52 ± 1.48 MPa; *p* < 0.001) when compared to the native group (6.68 ± 1.66 MPa).

**Figure 7:**
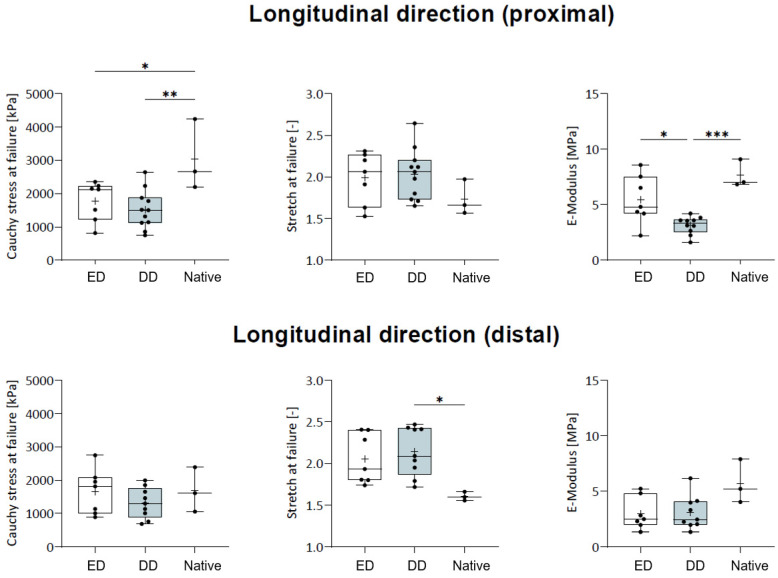
Comparison within urethra subregions in Cauchy stress, stretch at failure, and elastic modulus in the proximal and distal region of ED and DD group. E-Modulus: elastic modulus. (*—*p* ˂ 0.05; **—*p* ˂ 0.01; ***—*p* ˂ 0.001).

Further comparison within urethra subregions yielded a difference in Cauchy stress in the proximal region of the ED group (1772.6 ± 547 kPa; *p* = 0.031), DD group (1520.4 ± 551.2 kPa; *p* = 0.006), and the native group (3033.4 ± 873.0 kPa), respectively, but not for the distal regions (ED: 1660.1 ± 627.7 kPa; DD: 1315.2 ± 436.7 kPa; native 1686.0 ± 546.9 kPa; all *p* > 0.05, respectively (Figure 7). Comparison of the stretch at failure within the proximal (ED: 2.0 ± 0.3, DD: 2.0 ± 0.3, native: 1.7 ± 0.2) and distal (ED: 2.1 ± 0.3, DD: 2.1 ± 0.3, 1.6 ± 0.0) subregions yielded a significant difference only between the distal DD group and distal native group (*p* = 0.025), respectively. Similarly, the elastic modulus within the proximal (ED: 5.5 ± 2.1 MPa, DD: 3.5 ± 1.3 MPa, native: 7.7 ± 0.1 MPa) and distal (ED: 3.0 ± 1.1 MPa, DD: 3.1 ± 0.9 MPa, native: 5.7 ± 1.3 MPa) subregions only yielded a difference between the distal DD group and the native group, respectively (Figure 7).

**Figure 8:**
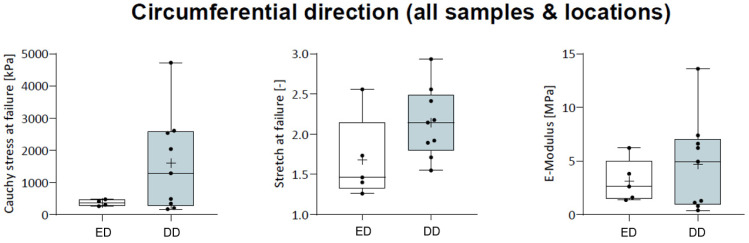
Circumferential load–deformation properties. Circumferential load–deformation properties are similar in both decellularization protocols. E-Modulus: elastic modulus.

Considering the mechanical properties such as Cauchy stress at failure (ED: 811.2 ± 969.4 kPa, DD: 1600 ± 1475.5 kPa), stretch at failure (ED: 1.5 ± 0.5, DD: 2.1 ± 0.4), and elastic modulus (ED: 3.1 ± 1.7 MPa, DD: 4.7 ± 4.2 MPa), no difference was observed between protocols (*p* > 0.05; Figure 8). However, group DD tended to demonstrate higher mean and median values, and the range of properties within the groups appeared to have a larger spread, as indicated by the minimum and maximum values.

### 2.5. Effect of Matrix-Conditioned Medium on Cell Viability 

**Figure 9:**
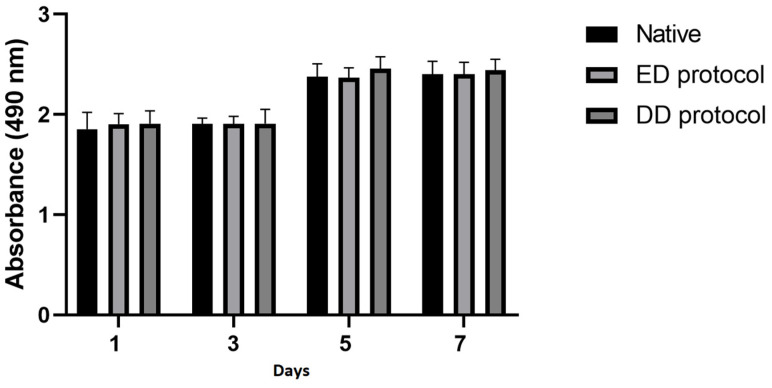
Proliferation assessed by MTT test results. Test sample absorbance values on specific days. Data are presented as the mean ± standard deviation. No significant decrease in the cell proliferation rate was detected when co-cultured with the matrix-conditioned medium.

The viability of adipose tissue-derived stem cells (ATSCs) was assessed using the MTT test on the first, third, fifth, and seventh days. Both control and matrix-conditioned medium supported cell growth. The matrix-conditioned medium did not exhibit toxicity, as evidenced by the absence of soluble toxins and no significant inhibitory effect on cell viability. However, statistical significance was not confirmed (Figure 9).

## 3. Discussion

The treatment of urethral disorders, such as injuries and strictures, has long been a significant challenge in urology. The current first-line treatment often involves the use of skin, preputium, skin flaps, oral or lingual mucosa autografts, and xenografts to replace damaged tissue [9]. However, using autologous tissue for urethral reconstruction poses several drawbacks, including high surgical trauma, donor site complications, and suboptimal restoration of anatomical and physiological function. Additionally, the issue of limited donor material remains unresolved [23,24]. Fortunately, advancements in TE have introduced promising new therapeutic strategies for urethral repair and reconstruction.

The decellularization of tissue is a well-known process that involves removing cellular components from a tissue or organ, leaving behind the ECM. The primary benefit is the preservation of the ECM composition and structural integrity, which is essential for cellular adhesion, migration, and differentiation. Preserved vascularization is crucial for the survival and function of the modified urethral tissue. Removing cellular components, foreign antigens, and inflammatory mediators can lower the recipient’s risk of immunological rejection [25]. However, it can be difficult to completely remove biological components; thus, residual DNA and proteins can provoke an immunological reaction. Decellularization can also lead to the loss of essential growth factors and bioactive molecules, hindering cellular processes and tissue regeneration. It may also weaken the tissues’ mechanical strength and cause structural alterations that could compromise tissue functionality [26].

In principle, there are three types of decellularization techniques for removing cellular components from tissues or organs while preserving the extracellular matrix. Each approach has its own advantages and disadvantages. Chemical decellularization (using detergents, acids, or bases) is relatively simple and effective at removing cellular components, but the harsh chemicals may damage the ECM and residual chemicals can provoke adverse reactions. Physical decellularization (such as freeze-thaw cycles, mechanical agitation, or osmotic shock) is less aggressive and causes less harm to the extracellular matrix, but it is often insufficient for complete cell removal. Enzymatic decellularization targets specific cellular components for removal but may degrade or alter the extracellular matrix. Combining various strategies to improve cell removal and enable customization based on tissue type and desired objectives appears to be the most effective approach [27].

Here, we present the comparison of two decellularization approaches, enzyme-detergent-based and detergent-based. Both led to the successful decellularization of the urethral tissue and to the preservation of the ECM architecture as well as the preservation of biomechanical properties. We applied these two protocols in order to find out whether the application of enzymes would lead to a significantly more effective removal of cells and their remnants. We found that there is no fundamental difference between the effectiveness of both approaches. A similar result was obtained by Simoes et al. [28], who used a chemical approach (they applied Triton X-100 and SDS solutions) in combination with mechanical agitation and perfusion to decellularize whole porcine urethras. In our previous study, we developed a protocol design, which was based on the enzyme-detergent-enzyme method. Trypsin and Triton X-100 were used to remove cells, followed by DNase treatment to remove DNA residues [29]. However, only a limited number of previous studies have addressed urethral decellularization to preserve the ECM components and the 3D structure of the urethral ECM. On the other hand, there are works that use other tissues. DeFilippo et al. [30] used tubularized porcine bladder for urethral reconstruction in rabbits. They used Triton X-100 and ammonium hydroxide for 14 days. Chun et al. [31] prepared acellular bladder submucosa matrix, in addition to Triton X-100, and they also applied trypsin in their protocol. In a more recent study, the MatrACELL decellularization process was used to prepare acellular placental membrane grafts for urethral replacement in a rabbit model. They used N-Lauroyl sarcosinate and endonuclease to minimize the impact of processing reagents on the biomechanical and biochemical properties of the tissue [32].

In our experiments, the dynamic approach (through agitation on an orbital shaker) was also utilized to promote homogeneous cellular removal from urethra tissue and thus increase the efficacy of the decellularization process. Other studies have also demonstrated that a combination of chemical and biological approaches with mechanical agitation leads to significantly removing cellular content and maintaining the main tissue architecture and properties [33,34].

Histochemical staining and scanning electron microscopy demonstrated that decellularization did not significantly alter the quantity or distribution of collagen, elastin, and fibronectin within the decellularized samples compared to the controls, indicating excellent preservation of the extracellular matrix. Given the limited capacity of most adult cells to produce elastin and modify elastic matrix structures, the preservation of collagen and elastin in decellularized tissue offers a significant advantage over alternative materials, such as synthetic polymers [35,36]. The well-preserved fibronectin, a crucial adhesion molecule facilitating cell adhesion, growth, and migration, combined with other glycosaminoglycans and the three-dimensional network of collagen fibers, are instrumental in enabling cells to infiltrate the scaffold, establish residence, and proliferate [37]. This cellular behavior is paramount for successful tissue regeneration.

Regarding the load–deformation properties of the tissues, it was found that no significant difference was observed between the ED and DD groups for Cauchy stress at failure, stretch at failure, and elastic modulus. DD tissues appear to have slightly less favorable tissue properties; however, the extent of the loss of mechanical tissue integrity appears to be subordinate. Moreover, predominantly, differences in tissue properties were more commonly observed in the proximal areas when loaded longitudinally, whereas sparse differences were detectable distally; there were not any detectable differences in the circumferential loading properties between decellularization protocols. Of note, the mechanical properties for longitudinal loading align well with those obtained in a previous trial using acellular porcine ureteric tissues [38]. Both the structural features and the biomechanical properties of native and acellular tissues are indicative of the decellularized tissues offering a similar construct to their native counterparts.

This study has several limitations that should be acknowledged. First, we lack long-term mechanical performance data, which limits our understanding of the durability of decellularized urethral tissues over extended periods. Additionally, while both protocols demonstrated efficacy in cell removal and ECM preservation, further optimization is required to enhance recellularization potential and minimize the impact on mechanical integrity for in vivo applications.

Future research should explore alternative decellularization agents that align with regulatory standards, especially for clinical applications. Enhancing our protocols for better integration and performance in clinical settings will be essential. Additionally, long-term studies assessing mechanical and biological performance are crucial to ensure the scaffold’s functionality and compatibility in regenerative medicine.

After overcoming the challenges and conducting further testing, particularly on animal models, decellularized urethras may be applicable mainly in regenerative urology. Decellularized urethral scaffolds could be instrumental in treating urethral strictures and defects by providing a structural matrix that supports the integration and proliferation of patient-specific cells to create customized grafts when needed, providing an adaptable and accessible solution for personalized treatment. These scaffolds, with preserved ECM components, may offer a natural biocompatible foundation for tissue repair and regeneration, potentially reducing the complications and limitations associated with traditional grafting methods. In addition, matrices prepared in this way can be stored long-term in biobanks, creating an accessible source of tissues for planned surgical procedures. Biobanking of decellularized urethras also supports ongoing research by providing a consistent source of high-quality scaffolds for studies on tissue engineering, cell–ECM interactions, and regenerative medicine protocols. This resource could be critical in developing new and improved methods for tissue regeneration.

## 4. Materials and Methods

### 4.1. Urethra Harvesting

Human urethra harvesting was conducted in accordance with current regulations and approved by the Institution for Health-Care Providers’ Surveillance Authority (permit no. 23/2022). Urethras from 21 male individuals (mean age 72 ± 12 years) were collected under antiseptic conditions from deceased adults 24–72 h postmortem, using a sterile permanent catheter as a guide wire. The skin was incised along the ventral side of the penis, from the subcoronal area to the base. Urethras, along with the corpus spongiosum, were surgically excised, averaging 7 cm ± 1 cm in length. The samples were then placed in a sterile collection solution containing 500 mg Edicin (Sandoz, Basel, Switzerland), 2 × 10^6^ U Penicillin (Biotika, Slovenska Lupca, Slovakia), 200 mg Clotrimazole (Ratiopharm, Ulm, Germany), and 240 mg Gentamycin (Sandoz, Basel, Switzerland) per liter of saline and immediately transferred to the laboratory, where they were stored at 4 °C for 3 h.

### 4.2. Decellularization Protocols

In total, 18 human urethra samples were divided into two groups. To decellularize the urethra, we looked at two approaches: the enzyme + detergent (ED) (*n* = 7) [21] and detergent + detergent (DD) (*n* = 11) protocol. The three remaining native samples served as controls. The steps are summarized in Table 1.

ED protocol—After 3 h in the collection solution, the samples were transferred to a new container with sterile deionized water (dH_2_O) with antibiotic antimycotic solution (Merck, Darmstadt, Germany) and washed on a shaker for 24 h at 4 °C to wash out erythrocytes. Subsequently, the samples were transferred to a new container with 0.25% Trypsin-EDTA (Merck, Darmstadt, Germany) solution and incubated with agitation on an orbital shaker at 4 °C for 24 h. After washing in dH_2_O for 2 × 10 min, the samples were placed in a new container with TRITON™ X-100 (Merck, Darmstadt, Germany) diluted 1:100 in dH_2_O and washed for 24 h at 4 °C with agitation on an orbital shaker. In the next step, after washing in dH_2_O, samples were incubated for 3 h at 37 °C in DNase I (200 μg/mL; Roche, Basel, Switzerland). Specimens were then placed in a sterile container containing dH_2_O, incubated at 4 °C, and relocated daily into a new container with dH_2_O for seven consecutive days.

DD protocol—After harvesting, the samples were transferred from the collection solution to a new container with dH_2_O for washing out. Subsequently, the samples were transferred to a new container with TRITON™ X-100 (Merck, Darmstadt, Germany) diluted 1:100 in dH_2_O and washed for 24 h at 4 °C with agitation on an orbital shaker. After washing in dH_2_O for 2 × 10 min, the samples were placed in a container with 1% SDS solution (Merck, Darmstadt, Germany) and washed for 24 h at 4 °C with agitation on an orbital shaker. After washing in dH_2_O, samples were incubated for 3 h at 37 °C in DNase I (200 μg/mL; Roche, Basel, Switzerland). Then, the samples were washed with dH_2_O and relocated daily as mentioned above under gentle agitation for seven days at 4 °C.

At the end of the decellularization process, several microbiological tests were routinely performed to guarantee tissue sterility in the protocol.

### 4.3. Light Microscopy and Immunohistochemistry (IHC)

We utilized the conventional formalin-fixed paraffin-embedded method to prepare untreated control specimens and decellularized tissue samples from the human male spongy urethra for examination under a light microscope. Standard processing procedures involved fixing the samples for 24 h at room temperature in 10% neutral buffered formalin (Centralchem, Bratislava, Slovakia), dehydrating them through a graded series of alcohols up to 100%, clearing them with xylene (Merck, Darmstadt, Germany), immersing them in paraffin (Merck, Darmstadt. Germany), and embedding them in paraffin blocks. Sections of 5 μm thickness were cut using a rotary microtome, then deparaffinized using xylene and rehydrated with alcohol concentrations decreasing to 50%. For visualization purposes, the sections were stained with hematoxylin and eosin (HE), Masson’s green trichrome, and orcein to highlight the cell nuclei, collagen fibers, and elastic fibers, respectively (all reagents sourced from Merck, Darmstadt, Germany). The stained sections were mounted on glass slides and analyzed with a LEICA DM2500 microscope (Leica Microsystems, Wetzlar, Germany), with photomicrographs captured using a LEICA DFC290HD digital camera (Leica Microsystems, Wetzlar, Germany).

For immunohistochemistry (IHC) analysis, antibodies against collagen IV and fibronectin (Merck, Darmstadt, Germany) were applied to detect different elements of the extracellular matrix (ECM). The PT Link system (Agilent Technologies, Santa Clara, CA, USA) facilitated the automation of pre-treatment stages, which included deparaffinization, rehydration, and antigen retrieval. Endogenous peroxidase activity was suppressed using a peroxidase-blocking reagent from the EnVision FLEX Detection System (Agilent Technologies, Santa Clara, USA) for 5 min. The primary antibodies were administered following the instructions provided by the manufacturer. Visualization of antibody binding was conducted using the EnVision FLEX Detection System with Diaminobenzidine (DAB) + Chromogen (Agilent Technologies, Santa Clara, USA). Cell nuclei were counterstained with Mayer’s hematoxylin for improved contrast within the untreated controls (Merck, Darmstadt, Germany). The slides were observed with a LEICA DM2500 microscope and representative images were captured using a LEICA DFC290HD digital camera.

### 4.4. Ultrastructural Analysis by SEM

For scanning electron microscopy, the native controls and decellularized tissue samples of human male penile urethras collected during an autopsy were fixed in 3% buffered glutaraldehyde (Merck, Darmstadt, Germany) for 4 h at room temperature. Afterward, the samples were rinsed three times in sodium phosphate buffer (pH = 7.3; Merck, Darmstadt, Germany) and postfixed in osmium tetroxide 1% solution (Merck, Darmstadt, Germany) at 4 °C. Next, the samples were gently dehydrated using an alcohol series of increasing concentrations up to 100% and dried at the critical point of CO_2_ using Critical Point Dryer LEICA CPD 300 (Leica Microsystems, Wetzlar, Germany). Then, the specimens were mounted on aluminum specimen stubs using carbon adhesive tapes. The non-conductive specimens were sputter-coated with a 15-nm thick gold/palladium layer in the LEICA EM ACE200 sputter coater (Leica Microsystems, Wetzlar, Germany) and observed by the ZEISS EVO LS 15 scanning electron microscope (Zeiss, Jena, Germany).

### 4.5. DNA Quantification and Qualitative Fragment Analysis

DNA isolated with the GeneJET Genomic DNA Purification Kit (Thermo Fisher Scientific, Waltham, MA, USA) and agarose gel electrophoresis was used to quantify and assess the quality of DNA in the decellularized urethras. Positive controls were native urethras (*n* = 3) of the same origin. In brief, native and decellularized urethral tissue was cut into small pieces and dried to a constant weight at a temperature of 37 °C. An accurately weighed sample was then resuspended in a digestion solution containing Proteinase K (Thermo Fisher Scientific, Waltham, USA) and incubated overnight at 56 °C. After incubation, RNase solution (Thermo Fisher Scientific, Waltham, USA) was added and allowed to stand for 10 min at room temperature. A lysis solution was then added and vortexed until a homogeneous suspension was obtained. After vortexing, 50% ethanol solution (Centralchem, Bratislava, Slovakia) was added, and lysates were transferred to a GeneJET DNA purification column (Thermo Fisher Scientific, Waltham, USA) and centrifuged. After two wash steps, the column was transferred to a sterile microcentrifuge tube, and elution buffer, which eluted the genomic DNA, was added. The concentration of the extracted DNA was measured spectrophotometrically using a NanoDrop spectrophotometer (Thermo Fisher Scientific, Waltham, USA) and the final amount of DNA (ng/DNA per mg/tissue dry weight) was calculated. For qualitative fragment length analysis, 10 μL of the total DNA was separated on a 1.5% agarose gel (40 min, 120 V). After the run, the gel was documented using the Azure C300 Imaging System (Azure Biosystems, Dublin, OH, USA).

### 4.6. Biomechanical Characterization

The specimens were then prepared for the biomechanical trials according to Scholze et al. [39,40], considering two experimental protocols. The first testing protocol entailed longitudinal load–deformation trials, and the second testing protocol circular load–deformation trials.

For the longitudinal load–deformation trials, dog bone-shaped samples measuring 20 × 5 mm were punched out of the opened urethral rings. The remaining tissues retrieved from the tapering were used for histological and ultrastructural analyses. Following this procedure, 22 samples were retrieved for the proximal urethra (*n* = 3 native, *n* = 6 ED group, *n* = 13 DD group) and 17 distal samples (*n* = 3 native, *n* = 6 ED group, *n* = 8 DD group).

For the circular load–deformation trials, ring specimens were harvested from the medial region (if applicable), depending on the length and integrity of the specimens. Tissue thickness was left unaltered in all samples. For storage purposes, all specimens and samples were precooled at 4 °C and frozen at −80 °C.

Prior to the experiments, all samples were osmotically adapted so to standardize their water content [41]. A protocol derived from ureter specimens was adjusted according to Schleifenbaum et al. [38].

The reference cross-sectional area of each sample was determined by analyzing scanned casts of the central specimen regions using dental impression materials (Panasil initial contact light, Kettenbach GmbH & Co. KG, Eschenburg, Germany) and the image processing package Fiji (an open source image processing package based on ImageJ, https://imagej.net/ij/). Before the tissues were mounted, a graphite speckle pattern was sprayed to track the local full-field tissue deformation [39,40]. To provide a better contrast, native specimens had to first be primed with white varnish [40].

The uniaxial load–deformation trials were conducted as outlined in Scholze et al. [39]. The mechanical experiments were conducted at 21 °C ambient temperature using a universal testing machine (20 kN Torsion Multi-Axis Testing System Z020) equipped with a 2.5 kN Xforce HP load cell (all ZwickRoell, Ulm, Germany). The samples were mounted to the pneumatic clamps of the testing device utilizing 3D-printed self-locking clamps and supporting arms. A digital image correlation system (Gesellschaft für Optische Messtechnik mbH, Braunschweig, Germany) was used at a sampling rate of 5 Hz for non-contact deformation recordings.

For the ring trials, customized L-shaped sample holders with stainless steel pins were 3D-printed and attached to the testing machine facing each other. Both longitudinal and circumferential tensile tests were performed using a quasi-static loading protocol consisting of 10 load–unload cycles between 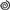 = 1.05 and 1.2 with a constant cross-head displacement of 10 mm/min (pre-conditioning), followed by an ultimate tensile test at the same displacement rate. Failure was defined as a sudden force drop of at least 30%.

### 4.7. Cytotoxicity Test

The MTT assay is a well-established method for assessing cell viability and cytotoxicity, which can be crucial for evaluating the biocompatibility of decellularized tissues of the urethra. The MTT assay is a colorimetric assay for assessing cell metabolic activity. NAD(P)H-dependent cellular oxidoreductase enzymes may, under certain conditions, reflect the number of viable cells in proliferation or cytotoxicity assay. These enzymes can reduce the MTT reagent to its insoluble formazan. The quantity of formazan product as measured by the absorbance at 490 nm is directly proportional to the number of living cell cultures.

First, 4 cm^2^ of decellularized urethral tissue was cut into small pieces, added to 50 mL of Dulbecco’s Modified Eagle Medium (DMEM, Merck, Darmstadt, Germany), and washed on a shaker for 24 h at 37 °C. After 24 h, the medium was filtered through a 0.2-μm filter, and 10% fetal bovine serum (FBS, Merck, Darmstadt, Germany) was added. For biocompatibility testing, we used ATSCs as they are multipotent and can differentiate into various cell types, making them a valuable model. ATSCs were seeded in a 96-well plate at a concentration of 5 × 10^3^ cells per well. The plates were incubated at 37 °C in a humidified atmosphere of 5% CO_2_ in the air for seven days. One plate was removed on days 1, 3, 5, and 7 to assess cell viability. Afterward, 20 μL of CellTiter 96 AQueosus One Solution Cell Proliferation Assay (Promega, Madison, WI, USA) was pipetted into each well, containing 100 μL of culture medium. The plates were incubated for 3 h at 37 °C. Absorbance was recorded at 490 nm using a 96-well plate reader BioTek EL 800 (Agilent, Vinooski, VT, USA). Unconditioned DMEM supplemented by 10% FBS was used as a negative control. All MTT tests were performed in sixplicates according to the standard protocol. The data are presented in the form of a graph.

### 4.8. Statistical Analysis

Data were analyzed using SPSS 14.0 (SPSS Inc., Chicago, IL, USA). The one-way analysis of variance with a Tukey post hoc test was used to determine significant differences. Data are presented as the mean ± standard deviation (SD).

Data processing was conducted using the MATLAB R2017b software (Mathworks, Natick, MA, USA). The crosshead displacement data obtained via the testControl II software provided mechanical parameters for all tested groups. Cauchy stress corresponds to the maximum stress of each group and the stretch at failure corresponds to the stretch observed at maximum force. The elastic modulus (Emod) was evaluated in the linear region of each stress–strain graph.

For statistical evaluation, Excel version 16.15 (Microsoft Corporation, Washington, DC, USA) and GraphPad Prism version 7 (GraphPad Software, Boston, MA, USA) were deployed. A between-group comparison was conducted using an ANOVA with Tukey post-hoc correction (native vs. ED group vs. DD group) or a two-tailed unpaired *t*-test (ED group vs. DD group), assuming a normal distribution of the data.

## 5. Conclusions

Urethral surgery and reconstruction pose significant challenges in terms of urethral patency after surgery, preventing urethral stricture recurrence, and patients’ quality of life. TE and regenerative medicine hold significant promise for transforming urological and surgical care by providing innovative solutions for tissue repair and replacement. Decellularization of the urethra for TE is a promising approach for regenerative medicine, offering potential treatments for urethral injuries and diseases. In summary, both decellularization techniques have significant potential in urethral tissue engineering but future research is needed to refine and optimize decellularization methods to improve scaffold recellularization and ensure clinical safety and efficacy. This paper might contribute to easier translation of TE techniques and research into clinical practice.

## Figures and Tables

**Table 1:** Summary of the decellularization methods.

ED Protocol		DD Protocol	
Solution	Temperature, Time	Solution	Temperature, Time
dH_2_O + ATB	24 h, 4 °C	dH_2_O + ATB	24 h, 4 °C
0.25% Trypsin-EDTA	24 h, 4 °C	TRITON™ X-100 1:100 in dH_2_O	24 h, 4 °C
dH_2_O	2 × 10 min, 4 °C	dH_2_O	2 × 10 min, 4 °C
TRITON™ X-100 1:100 in dH_2_O	24 h, 4 °C	1% SDS	24 h, 4 °C
dH_2_O	2 × 10 min, 4 °C	1% SDS	3 h, 37 °C
DNase I 200 μg/mL	3 h, 37 °C	dH_2_O	2 × 10 min, 4 °C
dH_2_O	7 d, 4 °C	DNase I 200 μg/mL	3 h, 37 °C
		dH_2_O	7 d, 4 °C

## Data Availability

Data are contained within the article.
